# Predictors of Acute Chest Syndrome in Patients With Sickle Cell Disease: A Cross-Sectional Observational Study

**DOI:** 10.7759/cureus.103552

**Published:** 2026-02-13

**Authors:** Urvin Patil, Sidharth S Pattnaik, Sailendra Nayak, Palash Das, Ambika Mohanty, Shubhransu Patro

**Affiliations:** 1 Internal Medicine, Kalinga Institute of Medical Sciences, Bhubaneswar, IND; 2 Internal Medicine, SCB (Srirama Chandra Bhanja) Medical College and Hospital, Cuttack, IND; 3 Pediatric Medicine, Kalinga Institute of Medical Sciences, Bhubaneswar, IND

**Keywords:** acute chest syndrome (acs), extravascular hemolysis, respiratory rate, risk predictors, sickle-cell disease

## Abstract

Background

Acute chest syndrome (ACS) is a major cause of mortality and hospitalization in patients with sickle cell disease (SCD). While clinical features often overlap with uncomplicated vaso-occlusive crises (VOC), early differentiation is critical for survival. Data regarding specific predictors of ACS in the Indian population remains limited. The objective of this study was to identify clinical and laboratory factors associated with the diagnosis of ACS in hospitalized patients with sickle cell disease.

Methods

This cross-sectional analytical study was conducted at a tertiary care teaching hospital in Eastern India between March 2023 and February 2025 and included 90 hospitalized patients with homozygous sickle cell anemia (HbSS). Patients were stratified into two groups: those diagnosed with ACS (n=33) and a control group of patients with uncomplicated VOC (n=57). Detailed clinical, hematological, and biochemical parameters were compared. Clinical and laboratory variables, including respiratory rate and lactate dehydrogenase (LDH), were analysed as early markers recorded at initial clinical presentation, prior to radiographic confirmation of ACS. Multivariate logistic regression and receiver operating characteristic (ROC) curve analysis were performed to identify independent predictors.

Results

The prevalence of ACS among hospitalized SCD patients was 36.67%. On univariate analysis, patients with ACS had significantly higher respiratory rates, heart rates, and inflammatory markers, total leukocyte count, C-reactive protein, and lactate dehydrogenase, along with lower hemoglobin levels compared to the non-ACS group (p < 0.05). Platelet counts did not differ significantly between groups. On multivariate logistic regression, respiratory rate showed an independent association with ACS (p = 0.001). ROC analysis demonstrated that a respiratory rate >21 breaths/minute had a sensitivity of 93.9% and specificity of 93% (area under the curve (AUC) = 0.944) for identifying ACS. LDH levels >389 U/L also demonstrated significant diagnostic discrimination (AUC = 0.769).

Conclusion

An elevated respiratory rate is a robust, accessible clinical parameter strongly associated with ACS in patients with SCD. Close monitoring of vital signs, particularly tachypnea, along with lactate dehydrogenase levels, may aid in early identification and risk stratification of ACS in resource-limited settings.

## Introduction

Sickle cell disease (SCD) is a chronic, inherited hemoglobinopathy that affects millions of individuals worldwide. It results from a single point mutation (Glu6Val) in the β-globin gene on chromosome 11, leading to the production of abnormal hemoglobin S (HbS). Under deoxygenated conditions, HbS polymerizes, causing red blood cells (RBCs) to assume rigid, sickled shapes. These cells exhibit reduced deformability, increased endothelial adhesion, and enhanced hemolysis, giving rise to the characteristic triad of vaso-occlusion, chronic hemolytic anemia, and progressive end-organ damage [1,2]. In India, SCD imposes a substantial disease burden, particularly among tribal and socioeconomically disadvantaged populations [3]; however, region-specific data on acute complications remain limited.

Acute chest syndrome (ACS) is defined as the presence of a new pulmonary infiltrate on chest imaging involving at least one lung segment, accompanied by symptoms such as fever, chest pain, cough, hypoxia, or respiratory distress [4]. ACS is the second most common cause of hospitalization in patients with SCD after vaso-occlusive crises (VOC) and is a leading cause of mortality in adults. Its pathophysiology is multifactorial, involving pulmonary infection, rib or sternal infarction, fat embolism secondary to bone marrow necrosis, and in situ pulmonary infarction due to sickling. These processes initiate a cycle of hypoxia, inflammation, and endothelial injury that can rapidly progress to acute respiratory failure if not identified early [2].

ACS is associated with significant short- and long-term morbidity. Acutely, it increases the risk of respiratory failure, prolonged hospitalization, and multi-organ complications, including neurologic events and acute kidney injury. Recurrent episodes contribute to chronic pulmonary sequelae such as restrictive lung disease, reduced pulmonary function, and pulmonary hypertension, which are associated with increased mortality [5]. It is clinically observed that in low- and middle-income countries such as India, ACS may be underdiagnosed or misclassified as pneumonia or tuberculosis due to overlapping clinical and radiographic features and limited access to early imaging and subspecialty care [4]. This diagnostic overlap can delay appropriate management and adversely affect outcomes.

Current management of ACS is largely reactive, relying on oxygen therapy, incentive spirometry, analgesia, antibiotics, and blood transfusion once the syndrome is established. Although disease-modifying therapy with hydroxyurea has been shown to reduce the frequency of ACS by decreasing VOC incidence and increasing fetal hemoglobin levels, its uptake remains suboptimal in many settings because of socioeconomic, educational, and healthcare access barriers, particularly in resource-limited regions [6]. Consequently, there is a need for additional strategies that enable earlier identification of patients at higher risk for ACS using readily available clinical and laboratory parameters.

Previous studies, predominantly from high-income countries, have identified several factors associated with ACS, including younger age, lower hemoglobin levels, leukocytosis, elevated inflammatory markers, prior episodes of ACS, comorbid asthma, and markers of increased hemolysis [7]. However, the diagnostic associations, relative contributions, and combined utility of these factors, especially as early indicators at initial clinical presentation, remain inadequately characterized, particularly in non-Western populations such as India.

In this context, the present study was conducted among hospitalized patients with SCD at a tertiary care center in eastern India. The primary objective was to identify clinical and laboratory factors diagnostically associated with ACS by comparing patients with ACS to those with uncomplicated VOC. By addressing region-specific gaps in evidence, this study aims to support earlier recognition and pragmatic risk stratification of ACS in routine clinical practice, especially in resource-limited settings.

## Materials and methods

Study design

This was a cross-sectional analytical study to evaluate clinical, laboratory, and radiological factors diagnostically associated with ACS in patients with SCD. The study also compared clinical and laboratory characteristics between patients with SCD who developed ACS and those admitted with uncomplicated VOC, and estimated the proportion of ACS among hospitalized SCD patients during the study period.

Study setting and duration

The study was carried out at a tertiary care teaching hospital, Kalinga Institute of Medical Sciences (KIMS), located in Bhubaneswar, Odisha, India, utilizing inpatient services of the departments of General Medicine, Pediatrics, and Hematology. The study duration was two years, from March 2023 to February 2025.

Study population

Eligibility Criteria

Patients with confirmed SCD presenting to inpatient services during the study period were screened for eligibility. Diagnosis of sickle cell disease was established using high-performance liquid chromatography (HPLC). To ensure diagnostic accuracy, patients classified as homozygous SCD (HbSS) on HPLC were further evaluated for the absence of hemoglobin A (HbA) and correlated with red cell indices and clinical phenotype. Patients of any age and gender with a confirmed diagnosis of HbSS were included after obtaining informed consent or assent, with guardian consent for minors. Patients with sickle cell trait or compound heterozygous conditions, including hemoglobin SC disease and hemoglobin S-β thalassemia, were excluded to maintain a homogeneous study population. Additional exclusions included pre-existing chronic pulmonary diseases unrelated to ACS, such as active or prior pulmonary tuberculosis, chronic obstructive pulmonary disease, bronchiectasis, or interstitial lung disease. Patients with asthma were included, as asthma is a recognized risk factor for ACS. Patients with recent COVID-19 infection, pregnancy, acute or chronic heart failure, severe impairment of consciousness, or need for intensive care or ventilatory support at the time of recruitment were excluded to reduce diagnostic overlap and confounding.

Classification

Patients were classified into two groups based on their clinical course during hospitalization. The ACS group included patients who fulfilled the National Acute Chest Syndrome Study Group criteria, defined as the presence of a new pulmonary infiltrate on chest radiograph involving at least one lung segment, along with one or more clinical features such as fever (≥38.5°C), a ≥3% decline in oxygen saturation from baseline, age-adjusted tachypnea, chest pain, cough, wheezing, rales, or signs of respiratory distress. This definition encompasses both infectious pneumonia and pulmonary infarction, which are clinically indistinguishable in the acute setting. The non-ACS group comprised patients admitted with uncomplicated VOC who did not have respiratory symptoms or new radiographic lung infiltrates at presentation and did not develop ACS during the course of hospitalization. Patients were followed throughout their hospital stay to determine whether ACS developed before final classification into study groups.

Sample Size and Sampling Method

All eligible patients admitted during the two-year study period were enrolled using consecutive sampling. No formal sample size calculation was performed. The sample size reflects the total number of hospitalized patients with HbSS meeting the inclusion criteria during the study period. The study was not designed to estimate population-level incidence but to explore diagnostic associations within a hospitalized cohort.

Data collection

All clinical and physiological variables, including respiratory rate, oxygen saturation, and chest examination findings, were recorded at the initial clinical assessment at presentation, prior to radiographic confirmation of ACS, and were analyzed as early presenting features rather than post-diagnostic criteria.

Data were collected using a standardized case record form and included demographic, clinical, laboratory, and radiological variables. Demographic and historical data included age, sex, residence, comorbidities, prior history of ACS or VOC, blood transfusion history, hydroxyurea therapy, smoking status, opioid use, and vaccination status. Clinical assessment included temperature, pulse, respiratory rate, oxygen saturation, blood pressure, and symptoms such as fever, chest or back pain, dyspnea, cough, wheezing, and rales. Laboratory investigations included hemoglobin, reticulocyte count, white blood cell count, platelet count, lactate dehydrogenase (LDH), bilirubin fractions, aspartate aminotransferase, alanine aminotransferase, and C-reactive protein (CRP). Hemoglobin fractions, including HbF, HbS, HbA₂, and HbA, were measured by HPLC. Chest radiography was performed in all patients, and high-resolution computed tomography (CT) was obtained when clinically indicated.

Statistical analysis

Data were analyzed using IBM SPSS Statistics for Windows, version 26.0 (IBM Corp., Armonk, New York, United States). Continuous variables were summarized as mean with standard deviation (SD) or median with interquartile range (IQR), depending on data distribution, and compared using Student’s t-test or Mann-Whitney U test as appropriate. Categorical variables were expressed as frequencies and percentages and compared using the chi-square test, with Fisher’s exact test applied when expected cell counts were less than five. Multivariate binomial logistic regression analysis was performed to identify variables independently associated with ACS. Statistical significance was defined as a p-value less than 0.05.

Prior to multivariate analysis, collinearity among candidate variables was assessed using correlation matrices and variance inflation factors. No variable demonstrated significant multicollinearity. Respiratory rate was retained in the final model due to its objectivity, continuous nature, and availability at initial bedside assessment, whereas symptom-based variables showed overlap and shared variance.

Ethical considerations

Ethical approval was obtained from the Institutional Ethics Committee of Kalinga Institute of Medical Sciences (approval number: KIIT/KIMS/IEC/1184/2023). Written informed consent was obtained from all participants or guardians. Data confidentiality was maintained through anonymization and secure data storage accessible only to study investigators.

## Results

A total of 90 hospitalized patients with HbSS were included in the analysis. Of these, 33 patients (36.67%) were diagnosed with ACS during hospitalization, while 57 patients (63.33%) were classified as having an uncomplicated VOC and did not develop ACS.

Baseline characteristics and clinical presentation

The study population predominantly comprised adults. The mean age of patients in the ACS group was 32.03 ± 11.69 years, compared to 32.39 ± 13.42 years in the non-ACS group, with no statistically significant difference between groups. Sex distribution was also comparable between the two groups.

Baseline demographic characteristics, presenting symptoms, vital parameters, and laboratory values recorded at initial clinical assessment are summarized in Table 1. Fever was the most frequent presenting symptom overall and was significantly more common in patients who developed ACS. Chest pain, cough, and shortness of breath were also significantly more prevalent in the ACS group. A history of non-invasive ventilation use in the preceding six months was observed exclusively among patients who developed ACS.

**Table 1:** Baseline demographic, clinical, vital, and laboratory characteristics at initial presentation in the study population Values are expressed as mean ± standard deviation or median (interquartile range), as appropriate. Categorical variables are expressed as number (percentage). All clinical, vital, and laboratory parameters were recorded at initial clinical assessment prior to radiographic confirmation of acute chest syndrome. Continuous variables were compared using Student’s t-test or Mann–Whitney U test, and categorical variables using Fisher’s exact test. A two-sided P value < 0.05 was considered statistically significant. ACS, acute chest syndrome; NIV, non-invasive ventilation; SpO₂, peripheral oxygen saturation; ALT, alanine aminotransferase; AST, aspartate aminotransferase; LDH, lactate dehydrogenase; CRP, C-reactive protein

Parameter	ACS Group (n=33)	Non-ACS Group (n=57)	p value
Age (years), mean±SD	32.03 ± 11.69	32.39 ± 13.42	0.821
Sex (male:female)	22:11	32:25	0.742
Fever, n (%)	25 (75.8%)	19 (33.3%)	<0.001
Chest pain n (%)	20 (60.6%)	11 (19.3%)	<0.001
Cough n (%)	17 (51.5%)	3 (5.3%)	<0.001
Shortness of breath n (%)	24 (72.7%)	6 (10.5%)	<0.001
History of NIV use (past 6 months) n (%)	6 (18.2%)	0 (0%)	<0.001
Heart rate (beats/min), mean±SD	109.7 ± 13.79	88.82 ± 10.76	<0.001
Respiratory rate (breaths/min), mean±SD	29.64 ± 6.86	17.75 ± 2.11	<0.001
Abnormal chest examination findings n (%)	31 (93.9%)	0 (0%)	<0.001
≥3% decline in SpO₂ from steady state n (%)	33 (100%)	0 (0%)	<0.001
Hemoglobin (g/dL), mean±SD	6.68 ± 2.03	8.15 ± 2.08	0.002
Total leukocyte count (/µL), median (IQR)	14,000 (7,320–21,845)	9,200 (6,795–13,550)	0.037
Platelet count (/µL), median (IQR)	236,000 (120,000–336,500)	190,000 (120,000–266,500)	0.503
Reticulocyte count (%), median (IQR)	6.19 (4.76–8.50)	4.40 (2.85–7.04)	0.032
Total bilirubin (mg/dL), median (IQR)	3.90 (2.08–6.00)	2.39 (1.56–3.85)	0.006
ALT (U/L), median (IQR)	48.6 (17.5–79.0)	34.0 (23.0–44.0)	0.264
AST (U/L), median (IQR)	70.0 (45.5–131.5)	67.0 (37.5–91.5)	0.172
LDH (U/L), median (IQR)	590 (344–956)	305 (201–446)	<0.001
CRP (mg/dL), median (IQR)	114.1 (54.15–183.35)	26.5 (10.2–62.85)	<0.001
HbS (%), mean±SD	70.24 ± 5.25	72.66 ± 5.32	0.040

Vital signs at presentation demonstrated marked differences between groups. Patients with ACS had significantly higher heart rates and respiratory rates compared to those without ACS. All patients in the ACS group exhibited a ≥3% decline in peripheral oxygen saturation from steady-state values at presentation, whereas none in the non-ACS group showed such desaturation. Abnormal chest examination findings were present in the vast majority of ACS patients and absent in the non-ACS group.

Laboratory parameters

Patients with ACS had significantly lower hemoglobin levels compared to those without ACS. Markers of hemolysis and inflammation were significantly elevated in the ACS group, including total leukocyte count, reticulocyte count, total bilirubin, lactate dehydrogenase, and CRP. Platelet counts and liver transaminases did not differ significantly between groups. Hemoglobin S percentage was modestly but significantly lower in patients with ACS.

Correlation analysis

Spearman’s rank correlation analysis demonstrated significant associations between ACS diagnosis and several early clinical, vital, and laboratory parameters, as shown in Table 2. Presenting symptoms such as fever, chest pain, cough, and shortness of breath showed moderate positive correlations with ACS. Vital parameters, particularly respiratory rate and heart rate, demonstrated strong positive correlations with ACS diagnosis. Hemoglobin level showed a weak but statistically significant negative correlation, while lactate dehydrogenase and CRP exhibited moderate positive correlations.

**Table 2:** Correlation of early clinical, vital, and laboratory parameters with acute chest syndrome diagnosis Spearman’s rank correlation was used to assess the association between early clinical, vital, and laboratory parameters and the diagnosis of acute chest syndrome. All parameters were obtained at initial presentation prior to radiographic confirmation of ACS. A two-sided P value < 0.05 was considered statistically significant. ACS, acute chest syndrome; SpO₂, peripheral oxygen saturation; ALT, alanine aminotransferase; AST, aspartate aminotransferase; LDH, lactate dehydrogenase; CRP, C-reactive protein.

Parameter	Spearman Correlation Coefficient	p value
Fever	0.409	<0.001
Chest pain	0.419	<0.001
Cough	0.536	<0.001
Shortness of breath	0.636	<0.001
Heart rate	0.751	<0.001
Respiratory rate	0.639	<0.001
Abnormal chest examination findings	0.953	<0.001
≥3% decline in SpO₂ from steady state	1.000	<0.001
Hemoglobin	−0.329	0.002
Total leukocyte count	0.221	0.037
Platelet count	0.071	0.506
Reticulocyte count	0.228	0.031
Total bilirubin	0.293	0.005
ALT	0.119	0.266
AST	0.145	0.174
LDH	0.449	<0.001
CRP	0.569	<0.001

Multivariate logistic regression analysis

To assess independent diagnostic associations with ACS, multivariate binomial logistic regression analysis was performed using variables that were significant on univariate analysis. The variables included respiratory rate, shortness of breath, chest pain, total leukocyte count, reticulocyte count, and hemoglobin level. The results of the regression analysis are presented in Table 3.

**Table 3:** Multivariate binomial logistic regression analysis for predictors of acute chest syndrome Multivariate binomial logistic regression was performed to identify independent predictors of acute chest syndrome using variables significant on univariate analysis. All variables were assessed at initial clinical evaluation prior to radiographic confirmation of ACS. A two-sided P value < 0.05 was considered statistically significant.

Variable	Adjusted Odds Ratio (aOR)	95% Confidence Interval	p value
Respiratory rate (per breath/min)	1.82	1.29 – 2.56	0.001
Shortness of breath	0.87	0.10 – 7.76	0.899
Chest pain	2.21	0.27 – 17.97	0.457
Total leukocyte count (/µL)	1.00	0.99 – 1.00	0.456
Reticulocyte count (%)	9998.1	0.001 – 1.97 × 10¹¹	0.283
Hemoglobin (g/dL)	0.72	0.44 – 1.17	0.186

Respiratory rate emerged as the only variable independently associated with ACS, with an adjusted odds ratio of 1.82 per breath per minute increase (95% confidence interval 1.29-2.56, p = 0.001), as shown in Table 3. None of the other variables retained independent statistical significance in the adjusted model. The lack of independent association for symptom-based variables such as shortness of breath and chest pain is likely attributable to collinearity with objective respiratory parameters.

Receiver operating characteristic (ROC) curve analysis

**Figure 1:**
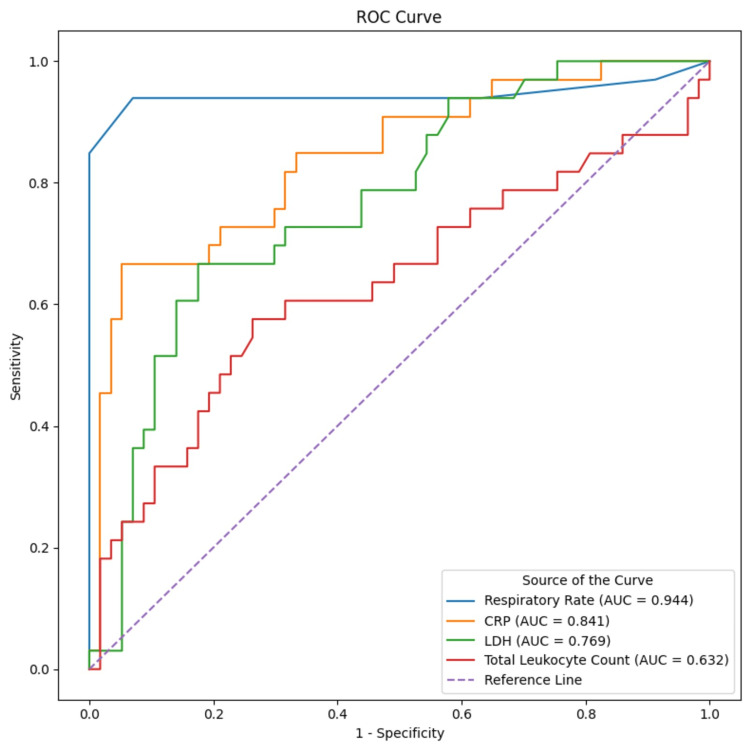
ROC curves for ACS diagnosis ROC, receiver operating characteristic; ACS, acute chest syndrome; CRP: C-reactive protein; LDH: lactate dehydrogenase

ROC curve analysis was performed to evaluate the diagnostic discrimination of selected clinical and laboratory parameters. The ROC curves for respiratory rate, total leukocyte count, LDH, and CRP are shown in Figure 1.

Respiratory rate demonstrated excellent diagnostic performance, with an area under the curve (AUC) of 0.944, while LDH showed moderate diagnostic discrimination with an AUC of 0.769. Based on ROC-derived thresholds calculated using Youden’s index, a respiratory rate cut-off of 21 breaths per minute yielded a sensitivity of 93.9% and specificity of 93% for identifying ACS. An LDH cut-off of 389 U/L demonstrated a sensitivity of 72.7% and specificity of 68.4%. These findings are illustrated in Figure 1.

## Discussion

This cross-sectional study examined hospitalized patients with SCD to explore early clinical and laboratory factors diagnostically associated with ACS at initial presentation. The objective was not to establish causality or prediction, but to identify pragmatic bedside parameters that may aid early recognition and risk stratification, particularly in resource-limited settings.

ACS remains a major complication of sickle cell disease, second only to vaso-occlusive crises as a cause of hospitalization and a leading contributor to intensive care unit admission and mortality [8,9]. Contemporary reviews continue to emphasize ACS as a key determinant of morbidity and survival in SCD, particularly in hospitalized adults [10]. Prior studies have shown that a substantial proportion of patients admitted with VOC subsequently develop ACS, often within the first few days of hospitalization [3]. While the proportion of ACS observed in the present cohort appears comparable to earlier reports, it should be interpreted strictly as a reflection of a hospitalized tertiary-care population rather than a population-level prevalence estimate [11].

Previous literature has identified several factors associated with ACS, including fever, hypoxemia, lower hemoglobin levels, leukocytosis, asplenia, and prior ACS episodes [7]. Other investigations, particularly among critically ill patients, have highlighted laboratory markers such as platelet count as indicators of disease severity and adverse outcomes [12,13]. In contrast, platelet counts did not differ significantly between groups in the present study. This discrepancy likely reflects differences in disease severity, study populations, and timing of assessment, as patients with severe disease requiring immediate intensive care support were excluded. These findings reinforce the heterogeneity of ACS and the context-dependent relevance of individual laboratory markers, as highlighted in recent expert reviews [14].

The most notable finding of this study was the strong independent diagnostic association between respiratory rate at presentation and ACS. Respiratory rate demonstrated the strongest association with ACS after multivariate adjustment, underscoring its value as an objective physiological marker of early respiratory compromise. However, it is essential to recognize that age-adjusted tachypnea is also included within established ACS diagnostic criteria [4,15]. Accordingly, respiratory rate should not be viewed as a standalone or causal predictor, but rather as an early clinical signal that may prompt closer monitoring and timely evaluation for evolving pulmonary involvement.

From a practical perspective, respiratory rate is inexpensive, universally available, and immediately measurable at the bedside. Its strong association with ACS highlights the importance of careful vital sign assessment in patients admitted with VOC, especially in settings where access to early imaging or subspecialty consultation may be limited. Elevated inflammatory and hemolytic markers, including LDH and CRP, further support the presence of pulmonary involvement, although they did not retain independent association after adjustment.

Several limitations must be considered when interpreting these findings. The cross-sectional design limits inference to diagnostic associations and precludes conclusions regarding temporal prediction. The modest sample size and single-center setting may restrict generalizability, and the exclusion of patients with severe disease requiring immediate ventilatory or intensive care support may have led to underrepresentation of the most severe ACS spectrum.

Despite these limitations, the study provides clinically relevant insights into early bedside parameters associated with ACS in hospitalized patients with SCD. Rather than proposing definitive predictors, the findings support the use of simple clinical measures, particularly respiratory rate, as adjuncts to clinical judgment in identifying patients who may warrant closer surveillance. Future multicenter prospective studies are needed to validate these associations.

## Conclusions

In this cross-sectional analysis of hospitalized patients with HbSS, ACS was associated with distinct early clinical and laboratory features at presentation. Among these, respiratory rate showed the strongest independent diagnostic association with ACS, underscoring its value as a simple and objective bedside parameter.

While respiratory rate overlaps with established diagnostic criteria for ACS, its strong association highlights the importance of meticulous monitoring of vital signs in patients admitted with VOC. Elevated markers of hemolysis and inflammation further supported the presence of pulmonary involvement but did not retain independent association after adjustment.

These findings should be interpreted as supportive diagnostic associations rather than definitive predictors. Larger, multicenter prospective studies are required to validate these observations and to determine whether integrating such parameters into structured assessment frameworks can improve early recognition and outcomes in patients with SCD.
